# Optimal PEEP Obtained by Titrating Inspiratory Oxygen Fraction Versus Electrical Impedance Tomography in Patients with High Risk of Intraoperative Atelectasis: A Randomized Controlled Trial

**DOI:** 10.3390/bioengineering13050533

**Published:** 2026-05-03

**Authors:** Lingling Gao, Lili Pan, Li Yang, Yu Cui, Jun Zhang

**Affiliations:** 1 Department of Anesthesiology, Fudan University Shanghai Cancer Center, Shanghai 200032, China; linglinggao@aliyun.com (L.G.); pllsyf@163.com (L.P.); liyanmagic@sina.com (L.Y.); 2 Department of Oncology, Shanghai Medical College, Fudan University, Shanghai 200032, China; 3 Department of Anesthesiology, UESTC Chengdu Women & Children Central Hospital, Chengdu 610091, China; cuiyun9831001@163.com

**Keywords:** positive end-expiratory pressure (PEEP), electrical impedance tomography (EIT), fraction of inspiratory oxygenation (FiO_2_), postoperative pulmonary complications (PPCs), robot-assisted laparoscopic prostatectomy (RALP)

## Abstract

Background: The optimal intraoperative positive end-expiratory pressure (PEEP) obtained by titrating to the lowest tolerable fraction of inspired oxygen (FiO_2_) has been proposed recently; however, whether its performance in obtaining optimal PEEP is comparable to that from electrical impedance tomography (EIT) titration remains unknown. Methods: Ninety-three adult patients undergoing robotic-assisted laparoscopic prostatectomy under general anesthesia were enrolled in this study. They underwent the determination of optimal PEEP obtained either by titrating to the lowest tolerable FiO_2_ (PEEP_O2_) or using EIT (PEEP_EIT_). The primary endpoint was intraoperative optimal PEEP values. Secondary endpoints included pre-extubation arterial oxygen partial pressure (PaO_2_)/FiO_2_, intraoperative mean arterial blood pressure (MAP), the incidence of hypoxemia in the postanesthesia care unit (PACU), and postoperative pulmonary complications (PPCs) up to discharge from hospital. Results: Group PEEP_O2_ (*n* = 47) exhibited a higher optimal PEEP compared to Group PEEP_EIT_ (*n* = 46) [Median (IQR): 18 (16–18 cmH_2_O) vs. 16 (14–16 cmH_2_O), *p* < 0.001]. Pre-extubation PaO_2_/FiO_2_ was higher in Group PEEP_O2_ (510.5 ± 80.0 vs. 471.8 ± 69.0 mmHg, *p* = 0.015), while lung dynamic compliance (41.1 ± 7.7 vs. 37.3 ± 6.4 mL cmH_2_O^−1^, *p* = 0.011) and static compliance (36.4 ± 5.8 vs. 33.6 ± 5.5 mL cmH_2_O^−1^, *p* = 0.017) were also higher in Group PEEP_O2_. Additionally, driving pressure (11.0 ± 2.0 vs. 12.1 ± 1.9 cmH_2_O, *p* = 0.006) was lower in Group PEEP_O2_. There were no significant differences in intraoperative MAP and the incidences of PACU hypoxemia and PPCs between the two groups. Conclusions: The optimal PEEP obtained by titrating to the lowest tolerable FiO_2_ is a clinically acceptable alternative of that obtained using EIT. Therefore, this technique could be a viable alternative to EIT for obtaining optimal PEEP.

## 1. Introduction

It is widely recognized that general anesthesia can lead to atelectasis, and certain surgical procedures and patient positioning can exacerbate this condition. Lundquist et al. reported that in 95 patients undergoing major abdominal surgery, 87% of the patients developed atelectasis [1]. Additionally, post-anesthesia pneumonia was reported, with incidences ranging from 0.5% to 28% [2], and atelectasis increases the risk of lung injury and pneumonia [3,4,5,6]. Fortunately, intraoperative atelectasis can be preventable or significantly reduced by administering appropriate positive end-expiratory pressure (PEEP), while minimizing intraoperative atelectasis can improve the outcome [7]. Therefore, it is essential to tailor the determination of optimal PEEP to each patient, taking into account factors like medical history, obesity, surgery type, intraoperative positioning, muscle paralysis, and pneumoperitoneum pressure [8,9,10]. However, determining the optimal PEEP level is challenging. The esophageal balloon has been used to determine the optimal PEEP [11]. This technique is not routinely used intraoperatively because special training is required; balloon malposition leads to inaccurate measurement of transpulmonary pressure and increased cost. Another non-invasive method to obtain optimal PEEP is to titrate driving pressure [12]. It does not reliably assess lung mechanics if the contribution of chest wall recoil counts for most respiratory system recoil. In patients with altered chest wall compliance (e.g., obesity or abdominal distension), a lower driving pressure may not reliably reflect the optimal PEEP necessary to counteract extrinsic forces compressing the lungs. It is an important variable but potentially problematic when guiding intraoperative ventilation [13]. Other limitations of driving pressure are well summarized in a recent review [14]. Electrical Impedance Tomography (EIT) offers a real-time, radiation-free method to visualize regional lung ventilation and identify the PEEP level that minimizes alveolar collapse and overdistension, and it has shown promise in reducing postoperative pulmonary complications [15,16]. However, its application can be challenging and unsuitable for certain types of surgeries. Furthermore, its availability is limited, and its cost-effectiveness has yet to be established. Therefore, there is a demand for the development of new practical techniques to determine and maintain optimal intraoperative PEEP.

Recently, a simpler alternative to EIT has been proposed: pulse oximetry-guided PEEP titration, involving the adjustment of PEEP and the fraction of inspiratory oxygen (FiO_2_) [17]. This technique is promising as it relies on a rationale: the best oxygenation must occur at the minimal intrapulmonary shunt, namely, the best oxygenation must co-exist with minimal atelectasis. Nonetheless, whether this method is safe and can offer comparable performance in identifying optimal PEEP and guiding intraoperative ventilation remains uncertain. In particular, this technique may not reliably detect excessive alveolar insufflation, which may cause lung injury. We hypothesized that the optimal PEEP obtained by titrating FiO_2_ to achieve the lowest but safe FiO_2_ or the best oxygenation was comparable to that obtained with the EIT method, a well-established technique to determine lung aeration [18]. We tested this hypothesis in patients at high risk of intraoperative atelectasis by using stepwise titrating PEEP. We also tested our second hypothesis that extubation at the optimal PEEP after surgery improved post-operative oxygenation. We aim to assess whether this simple technique poses no additional risk to patients and could be a viable alternative for optimal PEEP titration in such cases.

## 2. Methods

### 2.1. Ethics

This randomized controlled study was approved by the ethics committee of Fudan University Shanghai Cancer Center (FUSCC, Approval number: 2010225-11-2208A) and registered in the China Clinical Trial Registration Center (Identifier: ChiCTR2200062777; Website: https://www.chictr.org.cn/showproj.html?proj=176147 (accessed on 18 August 2022); principal investigator: Jun Zhang; Date of Registration: 18 August 2022). The subjects were recruited from 10 September 2022 to 12 December 2022 in FUSCC. An investigator (GL) assessed patients for eligibility the day before surgery. Written informed consent was obtained from each participant prior to the enrollment. All research was performed according to the relevant guidelines and regulations.

### 2.2. Inclusion and Exclusion Criteria

Male patients aged 18 years or older, American Society of Anesthesiologists physical status I–III, who were scheduled for elective robot-assisted laparoscopic prostatectomy (RALP) under general anesthesia with an endotracheal tube were recruited for this study. Patients with the following comorbidities were excluded: acute and chronic respiratory disorders, pulmonary hypertension, neuromuscular diseases affecting respiratory muscles, and/or preoperative SpO_2_ less than 95% on room air.

### 2.3. Anesthesia Management

The patients’ demographic and clinical characteristics were extracted from medical records. Intravenous access was established upon arrival at the operating room. Routine monitoring included electrocardiography, non-invasive blood pressure, SpO_2_, and core temperature. A radial arterial line was established to continuously measure arterial blood pressure and intermittent blood sampling for arterial blood gas (ABG) analysis. Patients were pre-oxygenated at an O_2_ flow rate of 6 L·min^−1^ until their expiratory oxygen concentration reached 80% or higher. Anesthesia induction was conducted with an intravenous targeted control infusion (TCI) of 4 μg·mL^−1^ propofol (effect-site concentration, Marsh mode) [19], 0.3 μg·kg^−1^ sufentanil, and 0.6 mg·kg^−1^ rocuronium. A 7.0-sized endotracheal tube was inserted, and correct placement was confirmed with capnography and auscultation. General anesthesia was maintained with a continuous TCI infusion of 3 to 4 μg·mL^−1^ propofol and 1 to 2 ng·mL^−1^ remifentanil (effect-site concentration, Minto mode). Under neuromuscular monitoring (TOF-Watch SX, Organon, Irish), intermittent rocuronium was administrated to maintain an intraoperative train of four = 0. A Narcotrend^®^ monitor was used to measure the depth of anesthesia, and the level was kept at a D0–D2 level. The surgical plethysmography index was used to estimate the analgesic level and adjust the use of remifentanil. All of the patients were treated with standardized fluid therapy, and infusion of balanced crystalloid fluid was adjusted according to intraoperative hemodynamics monitoring and clinical judgment of the care team. The infusion rate was 2–10 mL·kg^−1^ h^−1^ to maintain normal hemodynamics. If the mean arterial pressure < 65 mmHg, an intravenous bolus of 40 μg phenylephrine or 5 mg ephedrine was administrated if the heart rate was <50 bpm.

### 2.4. Interventions

After endotracheal intubation, the first recruitment maneuver (RM1) of 40 cmH_2_O for 15 s was performed, as described previously [17], and then mechanical ventilation was conducted with volume-controlled ventilation by using an anesthetic machine (Flow-I, Maquet Inc., Heidelberg, Germany). The ventilation was set at a tidal volume of 6 mL·kg^−1^ and a PEEP of 18 cmH_2_O first, and then the respiratory rate was to keep the end-tidal CO_2_ partial pressure between 40 and 50 mmHg. The pneumoperitoneum pressure was set at 12 mmHg, which is a routine practice in our center.

After the patients were placed in the Trendelenburg position and peritoneal insufflation was performed, they received the second recruitment maneuver (RM2), the same as RM1, followed by PEEP set at 18 cmH_2_O, considering that median individualized PEEP level ranges 12–16 cmH_2_O in laparoscopic surgery [10,19,20]. Then, all the patients underwent PEEP titration. For Group PEEP_O2_, FiO_2_ was initially set at 0.21, (1) if the SpO_2_ was at 95–96%, the optimal PEEP was 18 cmH_2_O; (2) if the SpO_2_ was greater than 96%, the PEEP was reduced by 2 cmH_2_O step-wise, with each step lasting for 5 min until SpO_2_ dropped below 96%, then the PEEP level set at this time was the optimal PEEP. If PEEP was reduced to 0 cmH_2_O while FiO_2_ was still kept at 0.21, FiO_2_ of 0.21 remained unchanged; (3) if the SpO_2_ was lower than 95%, the FiO_2_ was incrementally increased by 0.05 step-wise; each step lasted for 5 min to achieve a SpO_2_ of 95–96%. The PEEP level at the minimal FiO_2_ necessary to maintain a SpO_2_ of 95–96% was considered the optimal PEEP. The study was terminated if PEEP was increased to 18 cmH_2_O and FiO_2_ = 1.0 while the SpO_2_ remained lower than 95%. The detailed titration protocol was provided in Figure S1, as described in previous studies [17,20]. For Group PEEP_EIT_, the global and reginal lung ventilation were continuously monitored and recorded by EIT (PulmoVista500, Draeger Medical, Luebeck, Germany) [21]. In the present study, an EIT electrode belt, which carries 16 electrodes with a width of 40 mm, was placed around the thorax in the fifth intercostal space, and a reference electrode was placed on the right thorax. A customized PEEP titration also started from 18 cmH_2_O and decreased by 2 cmH_2_O step-wise for 5 min as described previously [10], during the room air ventilation. The optimal PEEP_EIT_ value was defined as the intercept of cumulated collapse and overdistension percentage curves to minimize regional compliance loss, which was analyzed by Costas algorithm using customized software. If the regional compliance decreases with the increase of PEEP, it indicates that the region may have excessive expansion. If the regional compliance decreases with the decrease of PEEP, it indicates that the region may collapse. We used changes in compliance to determine whether there are lung collapse and atelectasis. The individualized positive end-expiratory pressure titration process using electric impedance tomography was showed on Figure S2. The acquired optimal PEEP was kept constant throughout the procedure until extubation in both groups. The whole study protocol is shown in Figure S3.

The third recruitment maneuver (RM3) was conducted once the optimal PEEP was determined, as previously described [18]. The patients in both groups maintained the optimal PEEP after the third RM until extubation. During the intraoperative period, pulmonary ventilation by EIT was not recorded for either group due to the interference of the surgical electric cautery, and SpO_2_ ≥ 95% was maintained by finely adjusting the FiO_2_. Patients in both groups were extubated in the sitting position in the post-anesthesia care unit (PACU) once they met the criteria for extubation. Before extubation, the effect of rocuronium was reversed with intravenous 2–4 mg·kg^−1^ sugammadex.

### 2.5. Outcome Measurements

The primary endpoint was the intraoperative optimal PEEP value. Secondary endpoints included the arterial oxygen partial pressure (PaO_2_)/FiO_2_ before extubation, intraoperative mean arterial blood pressure, an accumulated dose of phenylephrine and ephedrine, the incidence of postoperative hypoxemia in the PACU defined as SpO_2_ less than 92% for 30 s on room air [22], and postoperative pulmonary complications (PPCs) identified before the discharge from the hospital.

Both groups obtained EIT data from a commercial EIT system (PulmoVista500; Draeger Medical, Luebeck, Germany). However, EIT data in Group PEEP_O2_ was recorded only while not used for PEEP titration. The EIT system also recorded the changes in regional lung ventilation in the two groups [23]. The “compliance win (CW)” and “compliance loss (CL)” were analyzed by using customized software. The “CW” and “CL” of the relatively selected reference interval were displayed, and their changes were presented in the form of percentage deviation. The change in regional compliance was regarded as the change in regional ventilation. The ABG results were obtained with a blood gas analyzer (GEM Premier 3500, Instrumentation Laboratory Co, MA, USA). Blood samples were collected to test whether the values of arterial blood oxygen saturation (SaO_2_) obtained from the ABG analysis were consistent with the timely corresponding SpO_2_ values recorded from the pulse oximeter. The data from 46 patients were compared with the Bland–Altman method, which is shown in Figure S3. The perioperative hemodynamic parameters and intraoperative respiratory mechanics data including pulmonary dynamic and static compliance (Cdyn and Cstat), driving pressure (defined as plateau pressure minus PEEP), and plateau pressure (determined as the pressure at the end of inspiration as displayed on the anesthesia machine), were continuously recorded. In the PACU, vital signs and ABG results were achieved at 5, 10, and 30 min after extubation, and supplementary O_2_ was provided to the patients via a nasal cannula if postoperative hypoxemia occurred. Based on consensus [24], PPCs are defined as respiratory infection, atelectasis, respiratory failure, bronchospasm, pleural effusion, and pneumothorax. The diagnosis of the PPCs was obtained by reviewing the medical records of the patients up to discharge from hospital to home.

### 2.6. Statistical Analysis

A previous study in the comparable patient population and for the same surgery showed that the median optimal PEEP_EIT_ was 14 cmH_2_O [10]. We assumed that a difference in PEEP levels between the two titration methods greater than 2 cmH_2_O would be clinically significant, the SD of each arm was assumed to be 3 cmH_2_O with an α error of 0.05 and a power of 80%, and at least 35 subjects were needed for each group. Considering a dropout rate of 28% based on our pilot study, 96 patients (*n* = 48 in each group at a ratio of 1:1) were enrolled in our study.

Randomization was conducted with MinimPy2 software (version 2.0, OSDN, Columbus, OH, USA), as previously described [25]. The randomization was performed after the patient entered the operating room by a study team member who was blinded to the trial protocol. Patients were stratified by age (<65 vs. ≥65 years) and BMI (<25 vs. ≥25 kg·m^−2^) to test differences in age and BMI distribution. An investigator (GL) generated the random allocation sequence, PL-enrolled participants, and assigned participants to interventions. All evaluators were blinded to the patients’ group allocation. Shapiro–Wilk test was used to check whether the variables were normally distributed. Continuous variables are presented as means ± SD or medians with interquartile range (IQR) according to whether the distribution was normal, while categorical variables are presented as counts and percentages. The unpaired *t*-test was used to compare demographic and clinical characteristics differences between the two groups. The Wilcoxon–Mann–Whitney test was used if the data were not normally distributed. Data collected at each time point were compared using repeated measures analysis of variance (ANOVA). Differences in the incidences of PACU hypoxemia and PPCs were assessed using the chi-square test. Statistical analysis was performed using SPSS 24.0 (IBM, Armonk, NY, USA) and GraphPad Prism version 8.0 (GraphPad Inc., San Diego, CA, USA). Statistical significance was set at *p* < 0.05.

## 3. Results

### 3.1. Clinical Characteristics

**Figure 1:**
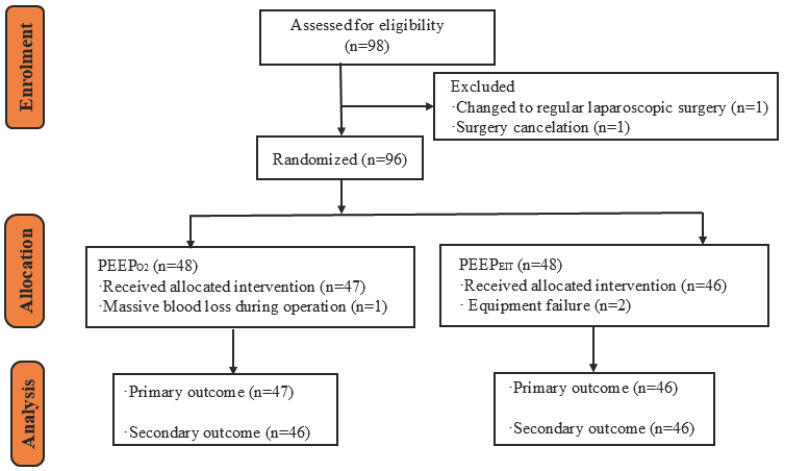
Flowchart of the study. PEEP, positive end-expiratory pressure; PEEP_O2_, optimal PEEP titrated with the step-wise reduction to identify the lowest in FiO_2_ as low as 0.21 to maintain SpO_2_ greater than or equal to 95%; PEEP_EIT_, optimal PEEP titrated with electrical impedance tomography; EIT, electrical impedance tomography.

A total of 98 patients were enrolled in this study. Two patients were excluded for operation cancellation or conversion to open surgery from the planned RALP. Therefore, 96 patients underwent the randomization. Three other patients were excluded after the randomization, including one for intraoperative massive blood loss and two for equipment failure. Thus, 93 patients were included in the primary outcome analysis. Since the data of one patient with 0 cmH_2_O optimal PEEP was missing immediately before and after extubation, this patient was excluded from the secondary outcome analysis but included in the final analysis for the primary outcome (Figure 1). The demographic and clinical characteristics of patients in the two groups were comparable, which is shown in Table 1, while the perioperative data are shown in Table 2.

### 3.2. Optimal PEEP Levels Between Two Groups

**Figure 2:**
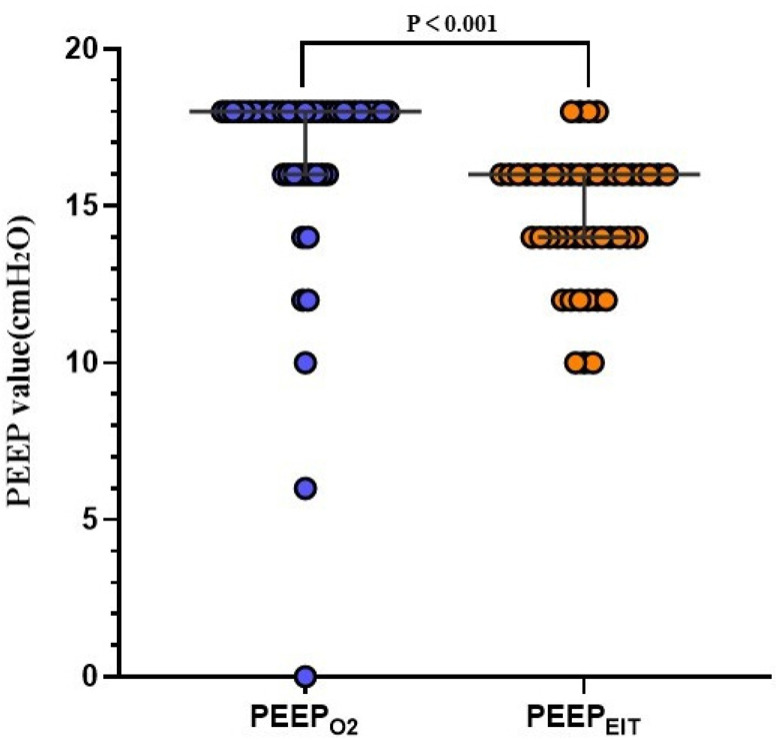
Comparison of optimal PEEP obtained with titration of FiO_2_ or EIT. PEEP_O2_, optimal PEEP titrated with the step-wise reduction in FiO_2_; PEEP_EIT_, optimal PEEP titrated with electrical impedance tomography. The data is presented as median with IQR for PEEP_O2_ (*n* = 47) and PEEP_EIT_ (*n* = 46) [Median (IQR), 18 (16–18 cmH_2_O) vs. 16 (14–16 cmH_2_O), *p* < 0.001].

The optimal PEEP level was significantly higher in Group PEEP_O2_ (*n* = 47) than in Group PEEP_EIT_ (*n* = 46) [Median (IQR),18 (16–18 cmH_2_O) vs. 16 (14–16 cmH_2_O), *p* < 0.001] (Figure 2).

### 3.3. FiO_2_, Oxygenation Status, and Ventilation Mechanics

**Figure 3:**
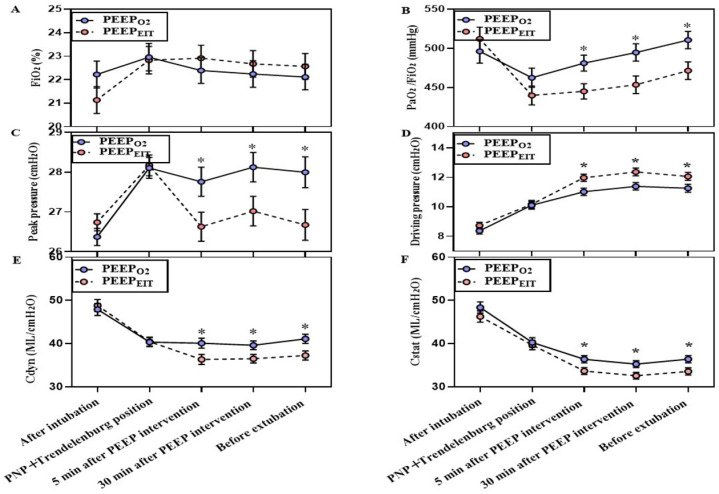
Time course of intraoperative oxygenation and respiratory mechanics. (**A**) Inspiratory oxygen fraction at different time points. (**B**) Arterial oxygen partial pressure/inspiratory oxygen fraction at different time points. (**C**) Peak pressure at different time points. (**D**) Driving pressure at different time points. (**E**) Lung dynamic compliance at different time points. (**F**) Lung static compliance at different time points. PEEP, positive end-expiratory pressure; PEEP_O2_, optimal PEEP titrated with the step-wise reduction in FiO_2_ as low as 0.21 to maintain SpO_2_ equal to or greater than 95%; PEEP_EIT_, optimal PEEP titrated with electrical impedance tomography (EIT). After intubation, immediately after intubation; 5 min after PEEP intervention, 5 min after setting the optimal PEEP; 30 min after PEEP intervention, 30 min after setting the optimal PEEP; before extubation, immediately after the surgery completion before restoring to the supine position; * *p* < 0.05. PaO_2_/FiO_2_, arterial oxygen partial pressure/inspiratory oxygen fraction; Cdyn, lung dynamic compliance; Cstat, lung static compliance. PNP, pneumoperitoneum.

There was no significant difference in intraoperative FiO_2_ across the overall study period between the two groups (Figure 3A). However, PaO_2_/FiO_2_ was significantly higher in Group PEEP_O2_ than that in Group PEEP_EIT_ at 5 min after PEEP titration (481.0 ± 70.7 vs. 445.2 ± 66.5 mmHg, *p* = 0.014), at 30 min after PEEP titration (494.7 ± 76.7 vs. 453.4 ± 72.2 mmHg, *p* = 0.010), and immediately after the surgery completion but before restoring to supine (510.5 ± 79.9 vs. 471.8 ± 69.0 mmHg, *p* = 0.015) (Figure 3B). The peak airway pressure was higher in Group PEEP_O2_ than in Group PEEP_EIT_ (Figure 3C), while the driving pressure was lower in Group PEEP_O2_ than in Group PEEP_EIT_ (Figure 3D). Both Cdyn (41.1 ± 7.7 vs. 37.3 ± 6.4 mL cmH_2_O^−1^, *p* = 0.011) and Cstat (36.4 ± 5.8 vs. 33.6 ± 5.5 mL cmH_2_O^−1^, *p* = 0.017) were higher in Group PEEP_O2_ than in Group PEEP_EIT_ immediately after the surgery completion but before restoring to supine (Figure 3E,F).

We also compared their hemodynamic parameters and found no significant difference between the two groups in intraoperative mean arterial blood pressure and heart rate at each time point (Table 3). The median accumulated doses of rescue drugs including phenylephrine [160 (0, 305) μg vs. 160 (30, 305) μg, *p* = 0.748] and ephedrine [6 (0, 12) mg vs. 6 (2.3, 12) mg, *p* = 0.378) were comparable between the two groups (Table 2).

In addition, we evaluated the regional lung ventilation at their optimal PEEP in two groups. There was no statistical difference in the proportion of lung overdistension between Group PEEP_O2_ and Group PEEP_EIT_ [Median (IQR), 8.0 (5.0–12.8) % vs. 7.0 (4.0–9.8)%, *p* = 0.098], while the proportion of lung collapse was significantly lower in the Group PEEP_O2_ than that in the Group PEEP_EIT_ [Median (IQR), 1 (0–3)% vs. 5.5 (3–8.8)%, *p* < 0.001]. The two groups had significant differences in compliance win and loss (Table 4).

### 3.4. Postoperative Hypoxemia and Pulmonary Complications

There were no significant differences in the incidences of hypoxemia between Group PEEP_O2_ and Group PEEP_EIT_ (4.3% vs. 6.5%, *p* = 1.00, Table 2) within 30 min after extubation in the PACU, and pulmonary complications (6.5% vs. 2.2%; *p* = 0.62, Table 2) at the time of discharge to home and the length of hospital stay [Median (IQR), 6.0 (5.0, 8.0) vs. 6.0 (5.0, 8.3), *p* = 0.671]. The data on the postoperative pulmonary complications is listed in Table S1.

## 4. Discussion

The main results of this study are as follows: (1) among patients with a high risk of intraoperative atelectasis, the optimal PEEP value titrated with the lowest tolerable FiO_2_ method was higher than that titrated with EIT (18 vs. 16 cmH_2_O) and absolute difference was 2 cmH_2_O or 11%; (2) the higher PEEP level in Group PEEP_O2_ co-exist with higher intraoperative PaO_2_/FiO_2_, lower driving pressure, and higher lung compliance; (3) there was no significant difference between the two groups in intraoperative hemodynamic instability and with comparable accumulated doses of rescue vasoactive drugs; and (4) the incidences of postoperative hypoxemia in PACU and pulmonary complications up to discharge to home were also comparable between the two groups. These findings provide an alternative optimal PEEP titration for intraoperative ventilation management in surgical patients with high risk of atelectasis.

Peritoneal insufflation with laparoscopic or robotic-assisted surgery in the Trendelenburg position is a well-recognized high-risk procedure leading to perioperative atelectasis [28]. This is a great model for testing the hypothesis that optimal PEEP in patients with healthy lungs, but a high risk of intraoperative atelectasis can be obtained with titrating PEEP against FiO_2_. We found that the difference in optimal PEEP between the two groups, PEEP_O2_ vs. PEEP_EIT_, was no greater than 2 cmH_2_O. Therefore, titrating FiO_2_ to obtain an optimal PEEP seems an alternative to the EIT-based method.

This study aimed to determine the optimal PEEP by titrating the lowest tolerable FiO_2_ to achieve a target SpO_2_ of 95% or higher, gradually adjusting the PEEP levels. This approach is grounded in basic respiratory physiology, as described before [17]. We assume that the optimal PEEP required to maintain the best SaO_2_ with the lowest but safe FiO_2_ reflects the minimal intrapulmonary shunt, assuming no intra-cardiac shunt. Our result also indicated that by increasing PEEP incrementally, the optimal PEEP could be obtained without excessive alveolar insufflation. The 2 cmH_2_O higher in optimal PEEP_O2_ value improved, rather than worsened, respiratory compliance with comparable hemodynamic stability. This means that the optimal PEEP obtained by using this method is at least equal to that obtained with EIT technique [20], and consistent with that in the previous study [29,30]. The difference of 2 cmH_2_O in optimal PEEP values is considered clinically insignificant in critical care settings [12,31]. In a perioperative setting, it should even be less clinically significant.

Optimal PEEP can be obtained using a variety of techniques. The gold standard for this purpose is a CT scan [29] and also is responsible for its popularity [18]. The limitation of this technique is also well recognized, mainly in terms of unfavorite cost-effectiveness. It exposes the patients to radiation and is not feasible to continuously assess lung aeration intraoperatively. However, titration of FiO_2_ to obtain the optimal PEEP possesses more advantages, including directly measuring oxygenation status, ease of adjusting the PEEP according to the requirement at the given stage of the surgery, and suitable for nearly any surgery as long as the patient is under general anesthesia and with tracheal intubation; more importantly, no extra-cost beside routine care. The disadvantage we encountered was that it took about 20 to 30 min to obtain the optimal PEEP and required lung recruiting. However, PEEP titration can be achieved parallel to the surgical process and does not require extra time. In addition, intraoperative atelectasis can be efficiently overcome by applying continuous PEEP without recruiting maneuvers [32]. The titration process seems complicated; however, the process can be easily performed using modern ventilators. Therefore, obtaining and maintaining optimal PEEP could be automated in future ventilator designs.

During the study, we encountered one patient whose PEEP_O2_ was zero cmH_2_O with FiO_2_ = 0.21, yet the patient maintained SpO_2_ above 95%, confirmed by ABG analysis. We interpreted this finding as a minimal intrapulmonary shunt even in the absence of PEEP for this particular patient in the Trendelenburg position and after pneumoperitoneum. The patients like this one are surely over-PEEPed if the recommended PEEP 14 cmH_2_O is applied [17]. Therefore, our observation implies the importance of individualized PEEP. Nevertheless, the median optimal PEEP obtained with FiO_2_ titration is consistent with that [18 (18–20) cmH_2_O] obtained in EIT in patients undergoing laparoscopic gynecological surgery in the Trendelenburg position and with pneumoperitoneum [33].

It has been a concern that the intraoperative optimal PEEP may not translate into postoperative benefit, as the rate of postoperative hypoxemia is not improved even when optimal PEEP is achieved and maintained intraoperatively [34]. This is likely because coughing at extubation may negate the benefits of optimal PEEP maintained during the operation. When patients cough with an endotracheal tube in place and without optimal PEEP, lung volume can decrease to the residual volume, significantly increasing the risk of atelectasis, regardless of the optimal PEEP created intraoperatively. Therefore, maintaining optimal PEEP intraoperatively provides the best intraoperative oxygenation and may reduce lung injury. However, the benefit can disappear due to coughing and the inability of the patient to close the glottis as they normally would, owing to the presence of the endotracheal tube. To address this issue, we designed our study so that patients were extubated in a sitting position with the presence of optimal PEEP obtained intraoperatively. We determined the incidence of postoperative hypoxemia by assessing the occurrence of hypoxemia in PACU. If the SpO_2_ is lower than 92% when the patient breathes room air for one minute, we classified it as postoperative hypoxemia. Because we only have two arms for this study, and the patients in both arms were extubated at the optimal PEEP in a sitting position, we could not assess the benefit of optimal PEEP in the postoperative period with certainty. However, we have historic data for comparison. In the same patient population, the incidence of postoperative hypoxemia is 30.4% with intraoperative fixed PEEP of 5 cmH_2_O even they were extubated in sitting position [19] vs. 4.3% (Group PEEP_O2_) and 6.5% (Group PEEP_EIT_) of the patients in this study who exposed to intraoperative optimal PEEP and were extubated at optimal PEEP.

The study has several limitations. First, the hypothesis was tested on patients who underwent RALP with healthy lungs, as this population is at high risk for developing intraoperative atelectasis and requires a high PEEP level to maintain adequate oxygenation [30,35]. Therefore, it is uncertain if our observations can be generalized to those patients with a low risk of intraoperative atelectasis and/or with compromised lung function, like the patients in critical care settings. Second, we must acknowledge that we did not perform intraoperative or postoperative CT scans, which are considered the gold standard for assessing lung aeration and atelectasis. As a result, we could not determine if the improved intraoperative oxygenation with optimal PEEP_O2_ translates to better outcomes, for oxygenation and lung compliance are surrogate markers, rather than direct, imaging-based measures of atelectasis. Our previous study has shown compared with a fixed PEEP = 5 cmH_2_O, that the PEEP_O2_ at least could significantly decrease the incidence of post-extubation hypoxemia in the PACU [19]. Therefore, our study provides valuable insights, and these limitations must be considered when interpreting the results, and further research with larger sample sizes and intraoperative CT scans is warranted to gain a more comprehensive understanding on the implications of PEEP_O2_ in other patient populations. Nevertheless, based on the physiological assessment, the 11% or 2 cmH_2_O greater optimal PEEP obtained with FiO_2_ titration is associated with better physiological variables and no decremental effect in short-term outcomes. Therefore, the difference in optimal PEEP between the two techniques is statistically but not clinically significant. Rather, titration of optimal PEEP via FiO_2_ leads to even better oxygenation and less under-aeration without risking excessive alveolar aeration or hyper-aeration. Third, there is a potential risk that we may have underestimated the optimal PEEP using EIT because our titration began at 18 cmH_2_O. This means we could not fully exclude the possibility that some individuals may have had an optimal PEEP higher than 18 cmH_2_O. However, we initiated titration at 18 cmH_2_O immediately after performing lung recruitment at 40 cmH_2_O. At 40 cmH_2_O, a healthy lung typically shows little to no collapse. In an ideal protocol, titration would start directly from 40 cmH_2_O, but maintaining such a high intrathoracic pressure poses a substantial risk of hemodynamic instability. For safety, we therefore selected 18 cmH_2_O as the starting point, which is also consistent with most published studies. Fourth, this was a single-center study. While this ensured protocol standardization, it may limit the external validity of our results. Furthermore, the nature of the intervention made blinding of the anesthesiologists performing the titration impossible, which could introduce performance bias. However, outcome assessors blinded to group allocation to mitigate detection bias.

## 5. Conclusions

The optimal PEEP titrated with the lowest tolerable and safe FiO_2_ method was 2 cmH_2_O (11%) higher than that obtained through EIT-based titration in patients with a high risk of intraoperative atelectasis. Higher optimal PEEP is associated with improved intraoperative oxygenation and respiratory mechanics, no compromised intraoperative hemodynamics and short-term outcomes, and no sign of hyper-aeration. The findings suggest that pulse oximetry-guided optimal PEEP is not inferior to that obtained with EIT-based titration. Considering its potential cost-effectiveness, this technique could be an alternative method for intraoperative individualized PEEP titration. Future studies involving larger and more diverse populations are warranted to evaluate long-term clinical outcomes of this technique, such as PPCs.

## Figures and Tables

**Table 1:** Baseline characteristics of the patients.

Variables	PEEP_O2_ (*n* = 47)	PEEP_EIT_ (*n* = 46)	SMD
Age (years)	66.9 (54–80)	68.2 (53–80)	0.22
<65, n (%)	18 (38.3)	13 (28.3)	0.21
Predicted body weight (kg)	65.5 (3.8)	65.8 (4.2)	0.08
BMI (kg m^−2^)	24.3 (2.7)	23.9 (2.8)	0.15
≥25, n (%)	27 (57.4)	24 (52.2)	0.10
ARISCAT score *			
>44, n (%)	3 (6.4)	2 (4.3)	0.09
ASA physical status, n (%)			
I	6 (12.8)	7 (15.2)	0.07
II	41 (87.2)	39 (84.8)	0.07
Smoking, n (%)			
Never	29 (61.7)	28 (60.9)	0.02
Former	9 (19.1)	10 (21.7)	0.06
Current	9 (19.1)	8 (17.4)	0.04
Hypertension, n (%)	13 (27.7)	11 (23.9)	0.09
Diabetes mellitus, n (%)	7 (14.9)	10 (21.7)	0.17
PaO_2_/FiO_2_ (mmHg) before intubation	402.5 (45.2)	407.5 (60)	0.09

The data are presented as mean (standard deviation, SD), mean (range), or numbers (%). PEEP, positive end-expiratory pressure; PEEP_O2_, optimal PEEP titrated with a step-wise reduction in FiO_2_ as low as 0.21 to maintain SpO_2_ greater than or equal to 95%; PEEP_EIT_, optimal PEEP titrated with electrical impedance tomography; BMI, body mass index; ASA, American Society of Anesthesiologists. * The ARISCAT score is used to predict the risk of postoperative pulmonary complications (PPCs) in patients undergoing surgery under general anesthesia according to seven perioperative variables, including patient age, preoperative SpO_2_, respiratory infection in the past month, preoperative anemia (Hb ≤ 10 g/dL), surgical incision, duration of surgery, and emergency procedure [26,27]. SMD: Standardized mean difference. A larger absolute value indicates a greater magnitude of difference between groups. Values of approximately 0.2, 0.5, and 0.8 represent small, moderate, and large differences, respectively.

**Table 2:** Perioperative clinical data.

Variables	PEEP_O2_ (*n* = 46)	PEEP_EIT_ (*n* = 46)	SMD	*p* Value
Blood loss (mL)	100 (100, 200)	200 (100, 200)	0.19	0.236
Urinary output (mL)	400 (300, 500)	375 (300, 463)	0.05	0.668
Total amount of fluid infusion (mL)	1858.7 (356.9)	1853.3 (337.7)	0.02	0.940
Administration of vasoactive agents				
Ephedrine (mg)				
Received medication, n (%)	31 (67.4)	35 (76.1)	0.19	0.354
Amount (bolus equivalents)	6 (0, 12)	6 (2.3, 12)	0.14	0.378
Phenylephrine (μg)				
Received medication, n (%)	28 (60.9)	35 (76.1)	0.33	0.116
Amount (bolus equivalents)	160 (0, 305)	160 (30, 305)	0.07	0.748
Anesthesia duration (min)	205.2 (38.4)	208.4 (33.4)	0.09	0.671
Surgery duration (min)	158.2 (33.4)	159.7 (27.4)	0.05	0.817
Optimal PEEP-value (cmH_2_O) *	18 [16,17,18]	16 [14,15,16]	0.99	< 0. 001
PaO_2_/FiO_2_ (mmHg) before extubation	510.5 (79.9)	471.8 (69.0)	0.56	0.015
Extubation time (min)	32.8 (4.1)	31.9 (4.7)	0.19	0.336
SpO_2_ < 92% with room air at PACU, n (%)	2 (4.3)	3 (6.5)	0.10	1.000
SaO_2_ after extubation at room air (%)	95.4 (2.3)	96.1 (2.0)	0.17	0.417
ICU admission, n (%)	0 (0)	0 (0)	-	-
Length of postoperative stay	6.0 (5.0, 8.0)	6.0 (5.0, 8.3)	0.13	0.671
Pulmonary complications up to discharge to home, n (%)	3 (6.5)	1 (2.2)	0.21	0.617
Respiratory infection	2 (4.3)	0 (0)	0.30	0.495
Atelectasis	1 (2.2)	1 (2.2)	0.00	1.000

The data are presented as mean (standard deviation, SD), median (IQR), or numbers (%); PEEP, positive end-expiratory pressure; optimal PEEP_O2_, PEEP titrated with the step-wise reduction inFiO_2_ as low as 0.21 to maintain SpO_2_ greater than or equal to 95%; PEEP_EIT_, optimal PEEP titrated with electrical impedance tomography; extubation time, the time from the patients arrived at PACU till extubation; SaO_2_: arterial oxygen saturation; ICU, intensive care unit. PACU, post-anesthesia care unit. * *n* = 47. SMD: Standardized mean difference. A larger absolute value indicates a greater magnitude of difference between groups. Values of approximately 0.2, 0.5, and 0.8 represent small, moderate, and large differences, respectively.

**Table 3:** Vital signs, respiratory and hemodynamic parameters at three time points during mechanical ventilation.

	PEEP_O2_ (*n* = 46)			PEEP_EIT_ (*n* = 46)			*P_w_*	*P_b_*
	After Intubation	5 min After PEEP Intervention	Before Extubation	After Intubation	5 min After PEEP Intervention	Before Extubation		
HR (bpm)	68.5 (8.7)	64.3 (7.5)	62.3 (6.2)	67.8 (8.0)	64.7 (6.0)	62.7 (8.1)	**<0.01**	0.756
MAP (mmHg)	82.1 (13.5)	79.6 (9.4)	82.2 (11.2)	79.2 (11.0)	82.9 (9.1)	82.5 (9.8)	0.337	0.086
Peak airway pressure (cmH_2_O^−1^)	26.4 (1.3)	27.8 (2.4)	28.0 (2.6)	26.7 (1.6)	26.6 (2.6)	26.7 (2.7)	**0.010**	**0.006**
Driving pressure (cmH_2_O^−1^)	8.4 (1.3)	11.0 (1.5)	11.0 (2.0)	8.7 (1.6)	12.0 (1.9)	12.1 (1.9)	**<0.01**	**0.006**
Cdyn (mL cmH_2_O^−1^)	47.1 (8.9)	40.1 (7.9)	41.1 (7.7)	48.8 (9.7)	36.3 (7.7)	37.3 (6.4)	**<0.01**	**0.022**
Cstat (mL cmH_2_O^−1^)	48.4 (9.2)	36.4 (5.7)	36.4 (5.8)	46.3 (8.4)	33.7 (5.4)	33.6 (5.5)	**<0.01**	**0.028**
PaO_2_/FiO_2_ (mmHg)	496.3 (92.5)	481.0 (70.7)	510.5 (79.9)	511.9 (90.7)	445.2 (66.5)	471.8 (69.0)	**0.001**	**0.016**
PaCO_2_ arterial (mmHg)	45.7 (4.4)	52.4 (7.4)	54.5 (8.1)	47.5 (5.8)	54.0 (7.3)	56.3 (9.2)	**<0.01**	0.947
PH	7.36 (0.04)	7.31 (0.04)	7.30 (0.05)	7.36 (0.04)	7.31 (0.05)	7.30 (0.05)	**<0.01**	0.539
BE (mmol L^−1^)	0.1 (2.2)	0.1 (2.1)	0.1 (2.1)	0.9 (1.7)	0.4 (2.0)	0.2 (2.1)	0.342	0.415
HCO_3_^−^ (mmol L^−1^)	25.1 (1.7)	24.4 (1.4)	24.1 (2.3)	25.6 (1.2)	24.9 (1.3)	24.5 (1.3)	**<0.01**	0.911
Hb (g dL^−1^)	12.8 (2.8)	12.0 (2.5)	11.3 (2.0)	12.9(2.7)	11.7 (2.5)	11.8 (2.3)	0.001	0.360

The data are presented as mean (standard deviation); after intubation, immediately after intubation; 5 min after PEEP intervention, 5 min after setting the optimal PEEP; before extubation, immediately after the surgery but before restoring to the supine position; *p*-values compare the two arms from a repeated-measures analysis of variance. P_w_: within-group interactions, P_w_ < 0.05, compared to the time point of after intubation, there is a statistically significant change as the surgical time progresses; P_b_: between groups interactions, P_b_ < 0.05, compared to the PEEP_EIT_, there is a statistically significant change as the surgical time progresses; PEEP, positive end-expiratory pressure; PEEP_O2_, PEEP titrated with the lowest FiO_2_ or 0.21 to maintain SpO_2_ greater than or equal to 95%; PEEP_EIT_, PEEP titrated with electrical impedance tomography; HR, heart rate; MAP, mean arterial blood pressure; Cdyn; dynamic compliance; Cstat, static compliance; BE, base excess; Hb, hemoglobin.

**Table 4:** Comparisons of regional ventilation distribution at optimal PEEP_O2_ and optimal PEEP_EIT_.

Variables (%)	PEEP_O2_ (*n* = 46)	PEEP_EIT_ (*n* = 46)	Wilcoxon Test	
			Z Value	*p* Value
CL HP	8 (5–12.8)	7 (4–9.8)	−1.653	0.098
CL LP	1 (0–3)	5.5 (3–8.8)	−4.845	<0.001
CW	0 (0–4.8)	6 (2–10)	−3.445	0.001
CL	0 (0–3)	6.5 (3.3–10.8)	−5.002	<0.001

The data are presented as median (IQR). PEEP_O2_, PEEP titrated with the lowest FiO_2_ or 0.21 to maintain SpO_2_ greater than or equal to 95%; PEEP_EIT_, PEEP value titrated with electrical impedance tomography; CL HP, compliance loss towards higher PEEP levels; CL LP, compliance loss towards lower PEEP levels; CW, compliance win; CL, compliance loss.

## Data Availability

The datasets used and analyzed during the current study are available from the corresponding author on reasonable request. The data are not publicly available due to [the original data involved in this study contains sensitive clinical patient information and personal privacy-related content].

## Supplementary Materials

The following supporting information can be downloaded at: https://www.mdpi.com/article/10.3390/bioengineering13050533/s1. Figure S1. Titration process of optimal PEEP guided by SpO_2_21 and EIT. SpO_2_, pulse oxygen saturation; RM1, first recruitment maneuver; PIP, peak inspiratory pressure; PEEP, positive end-expiratory pressure; PetCO_2_, partial end-tidal carbon dioxide pressure; EIT, electrical impedance tomography. Figure S2. Individualized positive end-expiratory pressure (PEEP) titration using electric impedance tomography (EIT). A–F: The different sections corresponding to different PEEP values (18–8 cmH_2_O, decreased by 2 cmH_2_O stepwise) during the titration process; G: Global impedance waveform; H: Tidal images; I: PEEP trial analysis; CL: compliance loss; The pixel compliance loss is calculated in relation to the maximum compliance which is ascertained within all sections. If there is a compliance loss towards higher PEEP levels (CL HP [%]), the loss is displayed in orange. If there is a compliance loss towards lower PEEP levels (CL LP [%]), the loss is displayed in white. The regions without a compliance loss are displayed in dark gray. Figure S3. The study protocol. PEEP, positive end-expiratory pressure; PEEP_O2_, optimal PEEP titrated with the step-wise reduction in FiO_2_ as low as 0.21 to maintain SpO_2_ greater than or equal to 95%; PEEP_EIT_, optimal PEEP titrated with electrical impedance tomography; RM, recruitment maneuver; PACU, post-anesthesia care unit. This protocol was adopted from Girrbach F et al.’s study and modified [10]. Table S1. Pulmonary complications at the time of discharge to home.The patient identifier was listed in the table.
