# Supplementary material for: Optimal PEEP Obtained by Titrating Inspiratory Oxygen Fraction Versus Electrical Impedance Tomography in Patients with High Risk of Intraoperative Atelectasis: A Randomized Controlled Trial
Source: Bioengineering (Basel). 2026 May 3;13(5):533. doi: 10.3390/bioengineering13050533 (PMC13203537; doi:10.3390/bioengineering13050533)
Supplement: Supplementary file 1 [file bioengineering-13-00533-s001.zip › bioengineering-4187940-supplementary.pdf]

Table S1. Pulmonary complications at the time of discharge to home

Pulmonary complications [n (%)]	$PEEP_{O2}$ (n=46)	$PEEP_{EIT}$ (n=46)
Respiratory infection	No.25	/
	No.31	/
Atelectasis	No.22	No.24
Respiratory failure	/	/
Bronchospasm	/	/
Pleural effusion	/	/
Pneumothorax.	/	/

Note: The patient identifier was listed in the table.

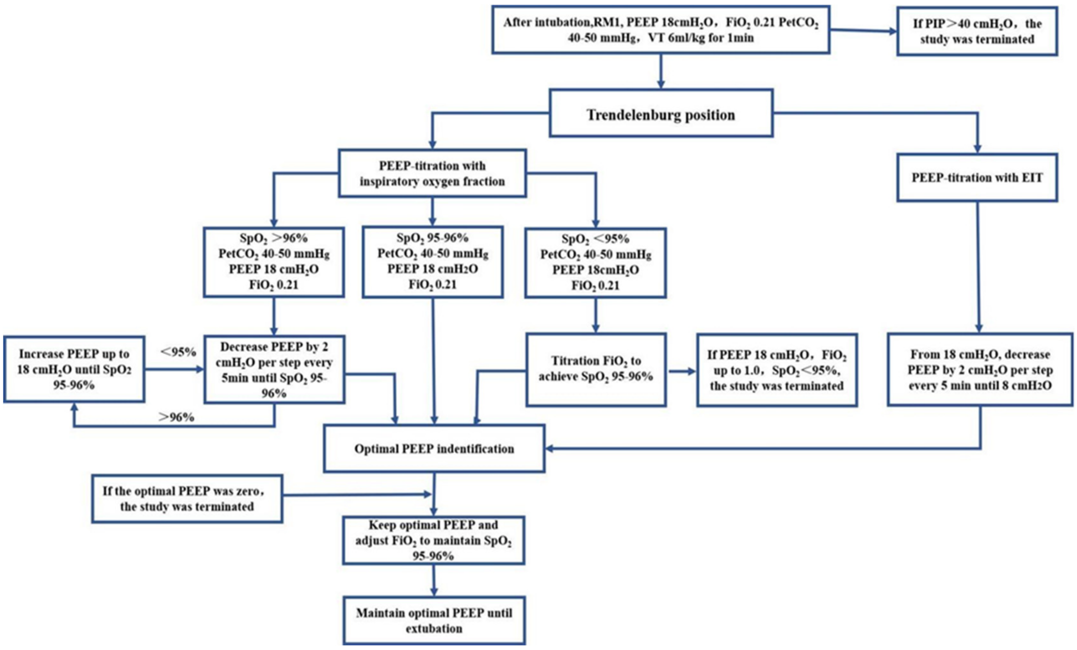

Figure S1. Titration process of optimal PEEP guided by SpO221 and EIT. SpO2, pulse oxygen saturation; RM1, first recruitment maneuver; PIP, peak inspiratory pressure; PEEP, positive end-expiratory pressure; PetCO2, partial end-tidal carbon dioxide pressure; EIT, electrical impedance tomography.

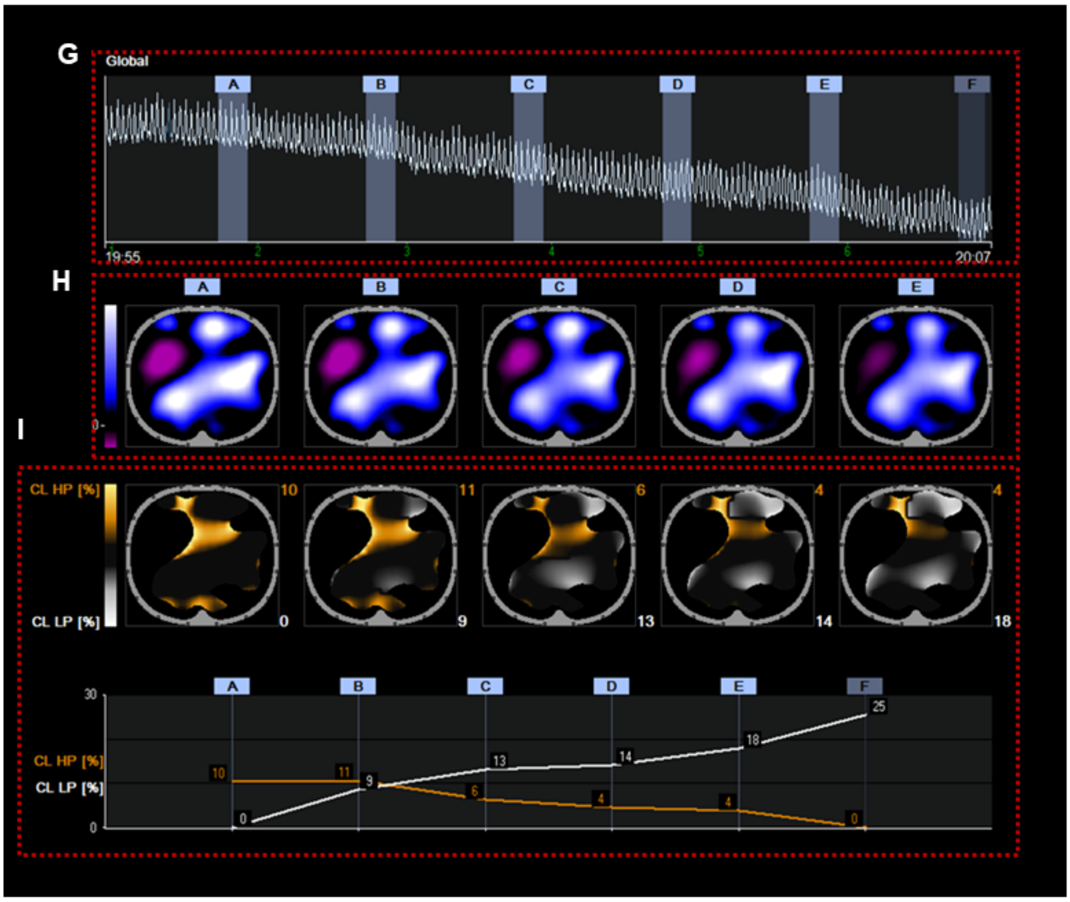

Figure S2. Individualized positive end-expiratory pressure (PEEP) titration using electric impedance tomography (EIT). A-F: The different sections corresponding to different PEEP values (18-8cmH2O, decreased by 2 cmH2O stepwise) during the titration process; G: Global impedance waveform; H: Tidal images; I: PEEP trial analysis; CL: compliance loss; The pixel compliance loss is calculated in relation to the maximum compliance which is ascertained within all sections. If there is a compliance loss towards higher PEEP levels (CL HP [%]), the loss is displayed in orange. If there is a compliance loss towards lower PEEP levels (CL LP [%]), the loss is displayed in white. The regions without a compliance loss are displayed in dark gray.

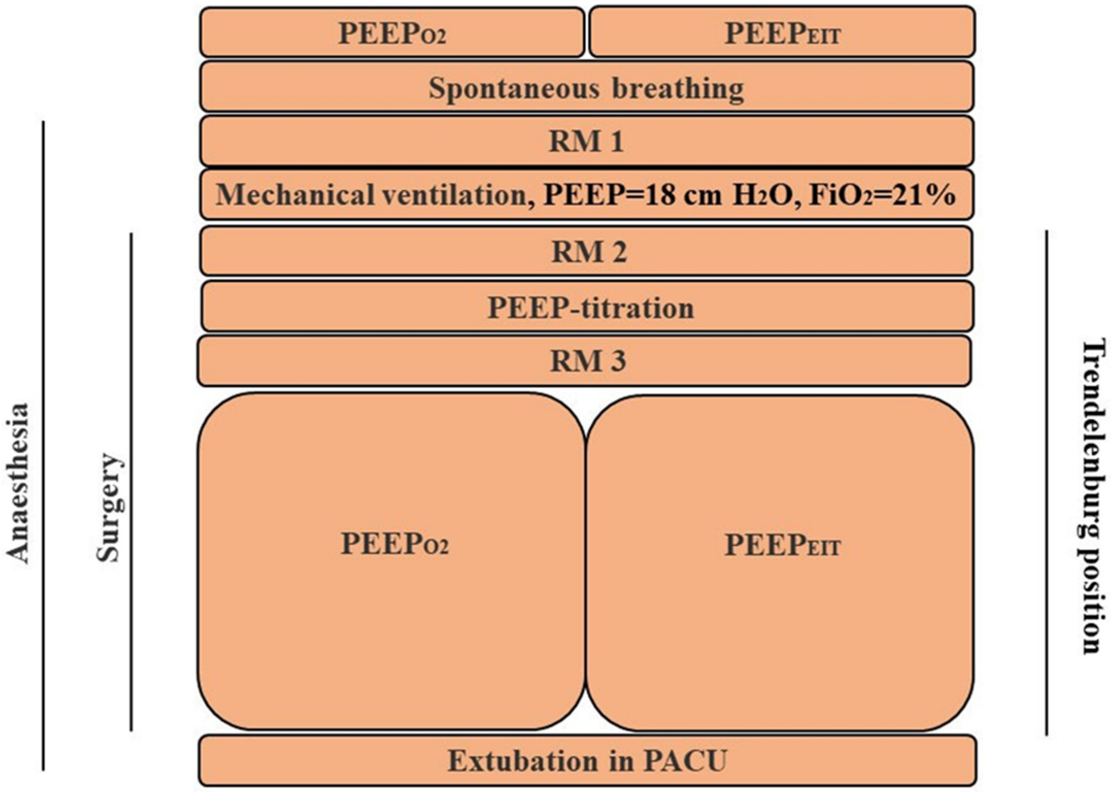

Figure S3. The study protocol. PEEP, positive end-expiratory pressure; PEEPO2, optimal PEEP titrated with the step-wise reduction in FiO2 as low as 0.21 to maintain SpO2 greater than or equal to 95%; PEEPEIT, optimal PEEP titrated with electrical impedance tomography; RM, recruitment maneuver; PACU, post-anesthesia care unit. This protocol was adopted from Girrbach F et al.'s study and modified [10].
